# Neurons in a Dish: A Review of In Vitro Cell Models for Studying Neurogenesis

**DOI:** 10.1111/jnc.70344

**Published:** 2026-01-09

**Authors:** Mariana Vassal, Ana C. Cruz, Sandra Rebelo, Filipa Martins

**Affiliations:** ^1^ Department of Medical Sciences, Institute of Biomedicine (iBiMED) University of Aveiro Aveiro Portugal

**Keywords:** cell models, differentiation, in vitro, neurogenesis, neurons

## Abstract

Understanding neurogenesis, the complex biological process of generating new neurons, is crucial for understanding brain development, function, and potential therapeutic interventions for neurological disorders. Due to the inherent difficulty of directly observing neurogenesis in the human brain, researchers heavily rely on cell models to simulate this process under controlled conditions. These models serve as invaluable tools to understand the mechanisms underlying the different stages of neurogenesis, helping researchers explore how neurons are generated, mature, and integrate into neural networks, thereby contributing to both normal brain function and neurological disorders. Therefore, this work provides a comprehensive overview of different cell models commonly used in neurogenesis research, from primary cultures and stem cells to immortalized cell lines. This compilation highlights the strengths and limitations of each cell model, which ultimately allows researchers to select the most appropriate model system for their research, thus enhancing the efforts towards unraveling the mysteries of the brain.

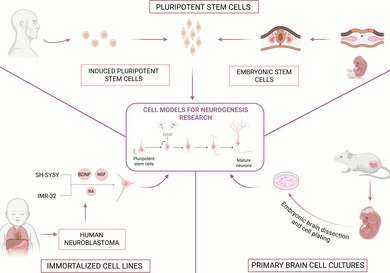

## Graphical abstract

Different in vitro cell models are valuable to study the different steps of neurogenesis, from the proliferation of neural stem and progenitor cells to the maturation of neurons. Pluripotent stem cells (including embryonic stem cells and induced pluripotent stem cells), immortalized human neuroblastoma cell lines (SH‐SY5Y, IMR‐32), and primary brain cell cultures each offer unique advantages and limitations for modeling neuronal development. While primary cultures closely mimic in vivo neuronal characteristics, neuroblastoma cell lines and pluripotent stem cells provide human‐derived and highly flexible alternatives that enable the study of specific neurogenic stages under controlled conditions.

**Abbreviations**
2D: two‐dimensional
3D: three dimensional
ASCL1: Achaete‐scute homolog 1
BDNF: brain‐derived neurotrophic factor
bFGF: basic fibroblast growth factor
BMP7: bone morphogenetic protein 7
cAMP: cyclic adenosine monophosphate
DG: dentate gyrus
DNA: deoxyribonucleic acid
EB: embryoid body
EGF: epidermal growth factor
ERK: extracellular signal‐regulated kinase
ESCs: embryonic stem cells
FGFs: fibroblast growth factors
HSPA14: heat shock protein family A member 14
iPSCs: induced pluripotent stem cells
MYCN: N‐myc proto‐oncogene
NCAM2: neural cell adhesion molecule 2
NGF: nerve growth factor
NSCs: neural stem cells
NTNG2: Netrin G2
PACAP‐38: pituitary adenylate cyclase‐activating polypeptide‐38
PDE5: phosphodiesterase type 5
PFDN6: prefoldin subunit 6
PKC: protein kinase C
RA: retinoic acid
RARs: retinoic acid receptors
SGZ: subgranular zone
SVZ: subventricular zone
TPA: 12‐O‐tetradecanoylphorbol‐13‐acetate
TPD52: tumor protein D52
TrkB: tropomyosin receptor kinase B

## 1 Introduction

Neurogenesis, the process of generating new neurons, was once thought to be limited to early brain development, a critical period during which new neurons populate emerging brain regions, establishing the neuronal circuits essential for brain growth and function (Urbán and Guillemot 2014). For many years, it was believed that once the brain matured, the generation of new neurons ceased. However, over the past few decades, research has shown that this process continues throughout life in restricted regions, such as the hippocampal subgranular zone (SGZ) within the dentate gyrus (DG) and the subventricular zone (SVZ) adjacent to the lateral ventricles, where it contributes to neural plasticity, learning, memory, and potentially repair following injury or disease (Jurkowski et al. 2020). Beyond these canonical niches, reports have proposed the existence of some neurogenic activity in other regions, including the hypothalamus, striatum, substantia nigra, cortex, and amygdala, although evidence in these areas remains inconsistent and often debated (Vassal et al. 2024). This evidence that neurogenesis persists in the adult brain highlights the critical need to characterize its underlying mechanisms, to understand how it contributes to brain function and to enable modulation of brain processes (Gideon et al. 2024).

The intricate process of generating new neurons from neural stem cells (NSCs) and their progression towards becoming mature neurons has been thoroughly detailed in numerous studies (Vassal et al. 2024; Ming and Song 2011). For simplicity, neurogenesis can be broadly divided into four key stages: (1) proliferation of neural stem and progenitor cells, (2) fate specification into neurons, (3) migration of newly formed neurons to their final destinations, and (4) their initial differentiation and maturation (Liu and Song 2016). Characterizing each one of these steps is fundamental for advancing our understanding of brain function and repair.

Of note, neurogenesis is part of an interconnected system, meaning that other non‐neuronal cell types, such as astrocytes, oligodendrocytes, microglia, endothelial cells, fibroblasts, and blood cells, play crucial roles in supporting neuronal function within the central nervous system (Araki et al. 2021). These cells contribute to the homeostatic microenvironment and regulate the neurogenic niche through interactions with NSCs, as well as through signals from the cerebrospinal fluid, blood vessels, and surrounding neural networks. These interactions collectively influence the states of quiescence, proliferation, and differentiation within the neurogenic niche (Llorente et al. 2022). While non‐neuronal components are critical to the broader context of neurogenesis, for simplicity, this review will focus exclusively on the neuronal lineage.

While significant progress has been made in characterizing neurogenesis, its underlying mechanisms remain incompletely understood, mainly due to the structural complexity and dynamic nature of the nervous system. While in vivo models remain widely used for these studies, they also present significant limitations, including the aforementioned complexity of the nervous system, high costs, the time‐intensive nature of animal experimentation, and interspecies variability (Yang et al. 2022). Additionally, ethical and regulatory restrictions on animal use have been limiting experimental flexibility, which has motivated the development and adoption of alternative approaches (Ferdowsian and Beck 2011). To address these limitations, different in vitro models have been developed to replicate specific aspects of neurogenesis in a controlled environment. Through a reductionist approach, these models simplify the process by reducing some of its complexities, allowing for a more focused investigation of individual stages of neurogenesis, while offering insights that are otherwise difficult to achieve in vivo (Azari and Reynolds 2016). Therefore, this review provides a comprehensive and methodologically oriented overview of the currently available in vitro cell models for studying neurogenesis, including primary cell cultures, immortalized cell lines, and pluripotent stem cell‐based systems, as well as different ways to maintain their culture. Each model provides unique insights, with varying degrees of biological relevance, scalability, and experimental flexibility, making them valuable tools in neuroscience research. By comparing the strengths and limitations of these models, researchers can make informed decisions about which cell types and culture conditions are the most appropriate for their research goals, promoting more robust and reproducible research in the field.

## 2 Cell Models in Neurogenesis Research

### 2.1 Primary Brain Cell Cultures

Primary cultures of the brain are commonly derived from the dissociation of brain tissue, yielding a heterogeneous population of cells that may include neurons, astrocytes, microglia, oligodendrocytes, neural stem and progenitor cells that closely mimic the cellular composition found in vivo (Sloan and Lanjewar 2021). Human primary cultures from fetal brain tissue can offer higher physiological relevance than rodent cultures (Darbinyan et al. 2013; Yu et al. 2004; Ray et al. 2014), but they are not widely available commercially, present ethical concerns, are poorly characterized, and are technically demanding (Ray et al. 2014). These ethical and practical limitations led research to rely on primary cell cultures derived from rodent brains, since from an ethics standpoint, this source is generally more acceptable than human fetal tissue. Rodents are widely used due to their accessibility and the availability of well‐established protocols, but their use still requires careful ethical justification, and all experiments must strictly comply with institutional and regulatory guidelines for animal research (Kiani et al. 2022). Primary brain cell cultures are particularly useful to study specific events of neurogenesis mainly because during embryonic brain development, cells are still differentiating, allowing researchers to study the transition from progenitor cells to fully differentiated neurons and to explore the molecular and cellular mechanisms involved in this differentiation process (Urbán and Guillemot 2014). However, embryonic tissues become increasingly heterogeneous over development, yielding distinct cell populations across dissections and stages (Figueres‐Oñate et al. 2021; Gritti et al. 2009). Accurate interpretation of the cellular composition and differentiation stages of each embryonic neurogenic site is crucial since the specific makeup of these tissues directly influences the cell populations that emerge in culture after their dissection (Gritti et al. 2009; Lattanzi et al. 2015). Without this knowledge, researchers risk misinterpreting experimental results.

Although primary cultures do not fully recapitulate neurogenesis, isolating and characterizing multipotent NSCs remain valuable for early‐stage mechanistic studies, allowing researchers to investigate the intrinsic mechanisms and extrinsic cues that regulate the transition from multipotent NSCs to neurons. And although these cells can be isolated from the early brain structures during the initial embryonic days of rodents (around E10.5 (Chen et al. 2017; Ou et al. 2022)), most studies use cells from slightly later developmental stages, such as E15.5, when brain regions are more clearly defined and yield higher numbers of viable cells suitable for culture (Zhou et al. 2020; Martins et al. 2017). During this period, some cells still remain in a proliferative, undifferentiated state (Ou et al. 2022), which is suitable to study early neuronal development and stem cell expansion. Additionally, at this developmental stage, many cells have not yet extended axons, which likely contributes to their structural integrity during dissection and enhances post‐isolation viability (Weinert et al. 2015). Notably, this period also coincides with the onset of lineage diversification, as stem cells begin producing both neuronal and glial precursors (Zhou et al. 2020; Ahlenius and Kokaia 2010; di Bella et al. 2021). In contrast to isolating multipotent NSCs from embryonic brain regions, some protocols provide valuable insights into isolating NSCs directly from the conventional neurogenic niches of adult rodents, offering a more valuable model for studying adult neurogenesis (Jaberi et al. 2021; Guo et al. 2012). But although NSCs are the gold standard for studying neurogenesis, some studies have reported that once cultured, these cells can regain glial traits or lose niche‐dependent plasticity, which reduces their relevance (Ma et al. 2009).

NSCs isolated from developing brains or adult neurogenic niches are considered ideal due to their undifferentiated state, which allows researchers to recapitulate the full process of neurogenesis. But instead, many studies rely on progenitor cells obtained from other brain regions like the hippocampus and cortex, often at more advanced stages of embryonic development, typically for practical reasons. This approach is both technically and biologically motivated: these regions are often more accessible, contain higher numbers of progenitors that are already partially committed to specific neuronal lineages, and are easier to dissect, which translates into higher numbers of viable cells, providing more reproducible results (Seibenhener and Wooten 2012). Additionally, studying region‐specific progenitors enables researchers to investigate how neurogenesis is influenced by the local niche, offering insights into both normal brain development and even region‐specific neuropathologies (Kim et al. 2009).

The process of neurogenesis is similar across neurogenic areas, but there are important regional differences in cellular mechanisms to consider. For instance, the cortex presents a more temporally segregated and predictable model, where neuronal production significantly declines after birth (Bond et al. 2020). In contrast, in the hippocampus, particularly in the DG, it continues at a high rate, with the first postnatal days marked by a high neuronal production. The ongoing neurogenesis in the hippocampus, which persists into adulthood, is crucial for memory formation and synaptic plasticity (Bond et al. 2022). Meanwhile, cortical neurogenesis is largely restricted to early developmental stages, reflecting the cortex's reliance on established circuits for sensory processing, motor control, and cognition (Mukhtar and Taylor 2018). Despite the persistence of hippocampal neurogenesis, earlier hippocampal dissections are often preferred for neurogenic studies, as postnatal brains contain more mature neurons and active synaptic plasticity (Seibenhener and Wooten 2012; Mynlieff 1997), which have a more limited capacity to recapitulate multiple events of neurogenesis. At this later stage, cells are typically less proliferative and exhibit a reduced capacity for differentiation (Cole et al. 2020). Nevertheless, the use of postnatal neurons has advantages over embryonic ones: they minimize animal sacrifice (the mother does not need to be sacrificed), are easier to genetically manipulate, and are especially useful for studying genetically engineered mice that are early postnatal lethal, as described in (Beaudoin et al. 2012).

Methods for isolating, plating, and maintaining cells in culture are similar despite the targeted brain region, but the specific trophic factors required to support cell growth and differentiation vary significantly depending on the specific cell type (Shabanipour et al. 2019). Quantitative reporting of these differentiation outcomes is limited, which hinders direct comparison across protocols and limits the selection of the most suitable protocol for enriching particular neuronal subtypes and its efficiency. For example, brain‐derived neurotrophic factor (BDNF) is required for the maintenance of cortical neuron size and dendrite structure (Gorski et al. 2003), and while it is also traditionally considered a main trophic factor for hippocampal neurons, the knockout of BDNF receptors (tropomyosin receptor kinase B, TrkB) has minimal effects on hippocampal neuron survival (Hu et al. 2023).

As previously mentioned, neuronal production peaks during the early stages of embryonic development (Zhang et al. 2023), but some protocols still prefer to use tissues from later embryonic days due to the smaller tissue size and greater difficulty of isolating earlier embryos (Seibenhener and Wooten 2012). Cortical and hippocampal cultures are typically prepared from E17–E19 rat embryos (Davaa et al. 2025; Kaneko and Sankai 2014) or E15–E16 mouse embryos (Pischedda et al. 2018; Sciarretta and Minichiello 2010), yielding mostly neurons that retain morphological features similar to those observed in vivo. At these stages, neuronal production is largely complete, ensuring the inclusion of newly differentiated neurons. The tissue collection timing contributes to greater cellular homogeneity, improving experimental consistency (Ahlenius and Kokaia 2010). Despite the near completion of cortical and hippocampal neurogenesis at these later stages, isolating neurons at these timepoints is still a valuable option for studying the last steps of neurogenesis, such as the differentiation and maturation process of these cells (Seibenhener and Wooten 2012; Sahu et al. 2019).

#### 2.1.1 Culture Configurations

Due to the complexity of neurogenesis, most research teams use simpler culture systems, such as two‐dimensional (2D) cultures, which remain the most widely used in vitro models. Beyond their cost‐effectiveness, the 2D environment is also compatible with a wide range of experimental tools, enabling precise manipulation and observation of individual cells. The planar configuration makes cells more accessible to molecular probes, genetic manipulation techniques, and other treatments, ensuring a consistent effect across the entire cell population (Zhang et al. 2022). This accessibility and simplicity make tracking specific gene expression patterns, signaling pathways, or morphological changes over time a lot easier. Additionally, different research teams have established the sequence of neuron development after plating, providing a step‐by‐step examination of how neurons develop, differentiate, and mature in vitro, detailing each phase of their differentiation—from initial outgrowth to axon and dendrite formation, branching, and maturation (Dotti et al. 1988; Govek et al. 2005; Banker 2018; Qian et al. 2022). By pinpointing at which stage neurons are in a controlled environment, researchers can more accurately investigate the specific molecular, genetic, or environmental factors that influence neuronal development and differentiation.

**FIGURE 1:**
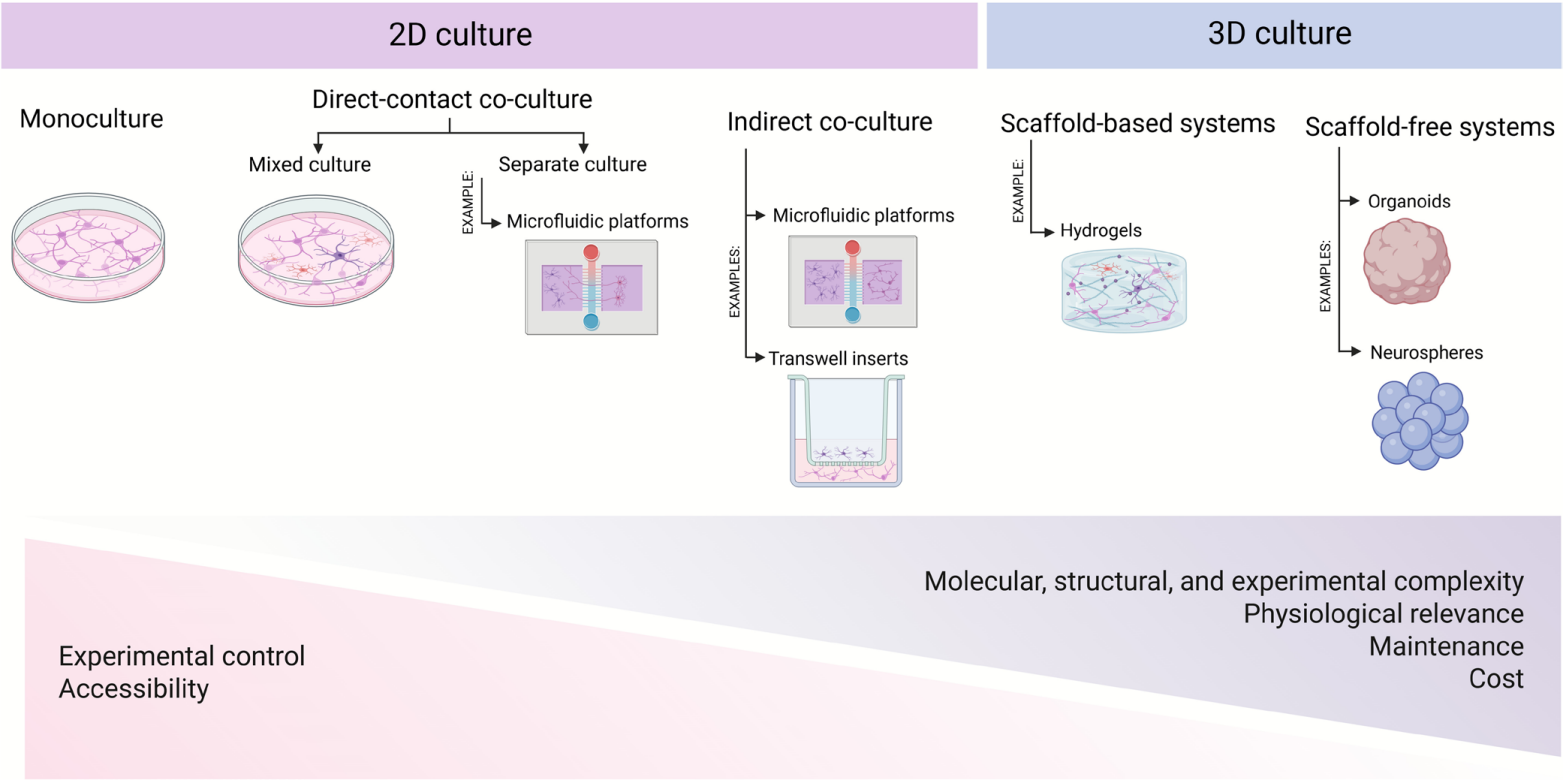
Comparative overview of in vitro culture systems for primary brain cultures. In traditional 2D cultures, primarily used for the culture of primary neurons, cells are grown on flat substrates, allowing for high experimental control and easy access to molecular tools and imaging techniques, though they offer limited physiological relevance (Centeno et al. 2018). Because these cultures largely consist of post‐mitotic neurons, they are more restricted in the developmental stages of neurogenesis they can model. Traditional 2D monocultures, typically composed of neurons alone, provide a simple and well‐controlled environment that facilitates molecular manipulation and imaging but lack the supportive cues and cellular diversity present in vivo (Zhang et al. 2022). Still within 2D systems, direct contact co‐cultures allow neurons and glia to coexist in the same environment and establish physical contact, either in mixed cultures, where all cell types share the same compartment, or in compartmentalized configurations, where a physical barrier separates the cell types while still allowing some level of contact between cell projections (such as microfluidic platforms with separate but connected chambers) (Liu et al. 2022). This latter approach has the advantage of mimicking in vivo neuron–glia interactions while controlling glia overgrowth (Shi et al. 2013). In contrast, indirect co‐cultures maintain neurons and glia in physically separated but fluidically connected compartments, allowing communication exclusively through soluble factors, as in porous membrane inserts (Transwell) or in some configurations of microfluidic platforms (Zhang et al. 2022; Yoo et al. 2023). Both types of compartmentalized co‐culture systems (direct or indirect) represent an improved 2D approach by enabling interactions between neurons and glial cells, which reduces glial overgrowth, enhances neurons' viability, and improves experimental reproducibility (Shi et al. 2013). In contrast, 3D configurations offer a more faithful representation of the in vivo environment, with enhanced structural and cellular complexity (Poli et al. 2019). By maintaining a stem cell population, these models are better at capturing multiple stages of neurogenesis. However, these models present greater technical challenges and increased variability. Created with Biorender.com.

Depending on the experimental aims, 2D cultures may be set up in different configurations: monocultures and co‐cultures (direct or indirect) (Zhang et al. 2022) (Figure 1).

As mentioned in previous sections, because the brain tissue contains different cell types at different stages, primary cultures often yield heterogeneous populations containing multiple cell types rather than pure neuronal populations (Soriano 2023). Over the years, numerous detailed protocols have been published, covering every step of the process, from tissue dissection at various embryonic stages to dissociating the tissue into single cells and growing them in vitro. Their popularity stems from their technical simplicity and the fact that neurons typically remain viable for 2–4 weeks (Darbinyan et al. 2013; Seibenhener and Wooten 2012; Sahu et al. 2019; Pacifici and Peruzzi 2012). Although culture longevity in standard primary rodent neuronal cultures is often limited to a few weeks in vitro, with substantial neuronal loss typically emerging after that time, specialized protocols can extend survival to 1–2 months (Kaneko and Sankai 2014), or even 5 months (Ray et al. 1993). However, these approaches are generally considered technically demanding, highlighting that extended maintenance is not yet routine. Quantitative reporting of neuronal survival and integrity over time is frequently lacking in the literature, complicating cross‐study comparisons and reproducibility, and should be addressed in future studies. Nevertheless, neurons in mixed cultures are typically more robust than in pure neuronal cultures due to the presence of glial cells, particularly astrocytes, which are essential for the survival and function of neurons (Aebersold et al. 2018). Additionally, this heterogeneity enhances the complexity of this type of culture, making it a more representative model that closely mirrors the cellular diversity and interactions observed in vivo (Barbosa et al. 2015). However, the use of mixed cultures introduces significant drawbacks, particularly in terms of variability, since the proportion of neurons to glial cells can vary between different dissections, resulting in cultures with different cellular compositions and potentially confusing experimental outcomes (Sahu et al. 2019). This variability is also further exacerbated by the frequent lack of quantitative reporting on neuronal‐to‐glial ratios. Incorporating this data in future studies is essential, as it allows researchers to assess culture composition, compare results across studies, and select the most suitable protocol for specific experimental questions. Such data can be obtained through simple immunocytochemical labeling of neurons (e.g., βIII‐tubulin) and glial cells (e.g., GFAP) combined with manual counting or automated image analysis (Lesslich et al. 2022). Standardized reporting of these parameters would greatly improve reproducibility and facilitate cross‐protocol comparisons.

Indeed, variations in glial cell content among different culture preparations can significantly impact neuronal behavior and function, as noted in (Ullian et al. 2001). This inherent variability complicates the reproducibility of experimental results and undermines the ability to draw consistent conclusions. Moreover, neurons lose their ability to divide once they differentiate, whereas glial cells, which outnumber neurons in some brain regions, retain the capacity to divide continuously in culture (Azevedo et al. 2009). This proliferative nature of glial cells can further interfere with or overshadow neuronal‐based observations (Murkherjee et al. 2023). To address these challenges, most of the available protocols are already optimized to selectively enrich neurons, often through an adhesion‐based selection (Sahu et al. 2019), through the use of specific growth factors (Ray et al. 1993), or through chemical treatments such as 5‐Fluoro‐2′‐deoxyuridine (Smith et al. 2023) or cytosine arabinoside (Seibenhener and Wooten 2012) that can arrest glial growth in cultures (Salazar et al. 2017). However, these strategies don't fully solve the variability issue; they only help to reduce it.

There are alternative techniques available that may help establish neuronal cultures composed entirely of neurons. Some studies describe the use of various neurocytometric methods to isolate and maintain neurons in culture, each offering distinct advantages and limitations, which will not be discussed in detail in this work. For reference, these include methods such as fluorescence‐activated cell sorting (FACS) (St. John et al. 1986), magnetic‐activated cell sorting (MACS) (Bowles et al. 2019; Holt et al. 2019), immunopanning (Barres 2014), among others. Although monoculture systems provide a controlled environment for studying neuronal development without the interference of other cell types, making them particularly valuable for mechanistic studies on neurogenesis, these cultures are not able to support long‐term survival (Aebersold et al. 2018).

Overall, it is clear that creating effective neuronal cultures presents a dilemma: while neurons require glial cells for survival (Aebersold et al. 2018), having a heterogeneous culture makes it difficult to control cell populations, which undermines experimental consistency (Smith et al. 2023). Without glial cells, neurons quickly deteriorate; with them, researchers lose the ability to ensure reproducible data exclusive from neurons (Lesslich et al. 2022). This unavoidable trade‐off complicates efforts to create ideal neuronal cultures for reliable research. Compartmentalized 2D co‐cultures have emerged as a promising approach to achieve this balance. These approaches involve using physical barriers to separate cell types, enabling the exchange of signaling molecules while maintaining limited or no direct cell–cell contact. The simplest approach involves transferring the supernatant (conditioned medium) from a separate glial culture to the neuronal cultures, but this feeder‐cell like system may be implemented with increasing levels of complexity (Liu et al. 2022). For instance, glial cells can be cultured on a coverslip placed inside the dish containing the neuronal cultures (Ioannou et al. 2019), or in a neighboring compartment, such as a porous membrane insert (Transwell) (Roqué and Costa 2017; de Simone et al. 2017). Other researchers have cultured astrocytes on a cellulose filter paper and suspended them above neuronal networks, allowing neurons to benefit from critical astrocyte‐secreted factors without direct contact, which improved neuronal viability and density (Aebersold et al. 2018). Microfluidic platforms are another obvious solution since they may allow not only to separate neurons from other cells, but also to isolate distinct segments of neurons, which is useful to study specific stages of neurogenesis, such as dendrite and axon formation (Park et al. 2009; Ristola et al. 2019).

Beyond enhancing neuronal survival and reducing variability between experiments, co‐culture systems also provide valuable insight into how other cell types impact neuronal properties (Jones et al. 2012). Of note, the presence of glial cells in co‐cultures has also been shown to improve neuronal transfection efficiency (Millet and Gillette 2012).

2D culture systems offer some unquestionable advantages, but they fail to capture the spatial organization and structural cues that are intrinsic to brain tissue. In vivo, the three‐dimensional (3D) architecture provides essential external signals that regulate proliferation, differentiation, migration, and circuit integration during neurogenesis (Yang et al. 2022). Consequently, more complex 3D culture systems are often required to more faithfully reproduce the brain's microenvironmental dynamics and functional complexity (Duval et al. 2017). It is well‐established that 3D culture systems offer numerous advantages over traditional 2D cultures, a concept that has been extensively explored in cell culture and tissue engineering for many years (Duval et al. 2017). While the general benefits of 3D cultures are widely recognized, this section aims to provide only an overview of the various 3D strategies available for culturing primary cultures and their potential to guide future research.

Primary neurons, along with other supporting cells, can be encapsulated within hydrogels, which form distinct 3D structures upon gelatinization, with both natural and synthetic hydrogels being currently used for this purpose. Besides providing a more physiologically relevant environment than conventional 2D cultures, their biomechanical manipulation allows researchers to control different cell behaviors including cell fate. For example, softer hydrogels tend to promote the differentiation of induced NSCs into neurons, whereas stiffer hydrogels encourage their differentiation into glial cells (Liang et al. 2021). A detailed analysis of how different hydrogel characteristics influence neuronal behavior can be found in (Madhusudanan et al. 2020). Despite these advantages, the use of hydrogels in 3D cultures poses challenges for imaging, limiting the ability to visualize cellular interactions and structures with high resolution (Shabanipour et al. 2019).

There are other scaffold‐free systems that also provide a physiologically relevant environment for studying different stages of neurogenesis. For example, neurospheres, also referred to as neural spheroids or neuro‐aggregates, are a classic method for culturing neurons in 3D, commonly used for studying the early stages of neurogenesis (Poli et al. 2019). In this cell culture configuration, neurospheres are formed by plating primary cultures from various brain regions at early stages of embryonic development, when NSCs are most abundant and active, or alternatively, by isolating the neurogenic niches of adult rodents, which still retain these NSCs (Ahlenius and Kokaia 2010; Khan et al. 2018). These heterogeneous free‐floating aggregates are composed of a variety of cells, including NSCs and progenitor cells, with some early differentiated neurons/glia, all embedded within a complex extracellular matrix organized in 3D (Ahlenius and Kokaia 2010). These cells are plated under non‐adherent conditions in a defined, serum‐free medium enriched with mitogens like epidermal growth factor (EGF) and basic fibroblast growth factor (bFGF), which support their viability and proliferation while preserving their undifferentiated state (da Silva Siqueira et al. 2021). An added advantage of this method is that the cells can later be transitioned back into 2D cultures for further experimentation (Das et al. 2019; Gonmanee et al. 2021).

Cells assembled in a neurosphere can be maintained with a high proliferation capacity, meaning they can be expanded and maintained for many passages (up to 60 (Gil‐Perotín et al. 2013)) in an undifferentiated state that can be differentiated into a neuronal lineage when desired, upon the removal of the aforementioned mitogens (da Silva Siqueira et al. 2021). After mitogen withdrawal, neuronal differentiation can occur within 10 days (Schwindt et al. 2009), with reported efficiencies varying widely depending on culture conditions. For instance, some studies have reported neuronal efficiencies as low as 10%, which can be increased up to 60% following exposure to valproic acid (Goffredo et al. 2008; Hsieh et al. 2004).

This simple culture method offers an effective platform for studying the behavior of NSCs, to monitor factors that may trigger the production of neurons, and to test their response in different settings (da Silva Siqueira et al. 2021; Ávila‐González et al. 2020), useful for studying neuronal fate specification (Gil‐Perotín et al. 2013). An added advantage is the availability of multiple optimized protocols for generating neurospheres from both embryonic and adult origins (Ahlenius and Kokaia 2010; da Silva Siqueira et al. 2021; Ávila‐González et al. 2020). For a better understanding of this technique, and prior to conducting any experiment, researchers should refer to the Silva Siqueira et al. review article that makes a comprehensive analysis of each step of the neurosphere assay protocol (da Silva Siqueira et al. 2021). Despite its broad use, many variables (which are reviewed in detail in (Gil‐Perotín et al. 2013)) influence the heterogeneity in cell composition within the neurospheres, which affects reproducibility between assays, posing challenges for its widespread application.

In addition to neurospheres, other scaffold‐free, self‐organizing structures have proven valuable for modeling neurogenesis, such as brain organoids, which have a much higher structural complexity and cellular diversity than neurospheres (Poli et al. 2019). This level of cell organization cannot be achieved using neurons, as they lack the stemness and self‐organizing capacity of pluripotent stem cells. However, a recent study demonstrated that mouse hippocampal brain organoids could be generated from primary neuronal cultures. This was achieved by enriching the culture with NSCs isolated from the SGZ of C57BL/6J mice at E14.5, a developmental stage that has high neuronal production in mice (Ciarpella et al. 2023). Despite this exception, pluripotent stem cells are typically the standard starting material for this type of cell culture configuration, which will be further discussed in Section 2.3.1.

### 2.2 Immortalized Cell Lines

As previously discussed, while primary cultures closely resemble the physiological properties of neurons and stem cells in vivo, they have several limitations. Primary neurons, in particular, have a finite lifespan, slow growth rate, and face the potential loss of their phenotype over time in culture (Slanzi et al. 2020). Additionally, although DNA can be introduced into these cells using specialized protocols (Sariyer 2013; Wollebo et al. 2013), they are generally more resistant to genetic manipulation (Martin et al. 2022). Lastly, ethical considerations regarding animal experimentation also limit their use. To overcome these challenges, researchers often use cell lines from neuronal tissues that became immortalized, whether as a result of their cancerous origin or their induction through specific treatments and genetic modifications (Gordon et al. 2013).

Many human and rodent immortalized cell lines of distinct origins are capable of acquiring a neuronal phenotype, including embryonal carcinoma cell lines (e.g., P19 (Yao et al. 1995), F9 (Alonso and Breuer 1991), NTERA‐2 (Amini and White 2021)), pheochromocytoma cell lines (e.g., PC12 (Xie et al. 2023)), and cell lines of neuronal origin (e.g., SH‐SY5Y (Kaya et al. 2024), IMR‐32 (Sharma et al. 2021), B35 (Otey et al. 2003), CAD (Li, Hou, et al. 2007), HT22 (Liu et al. 2009), C17.2 (Lundqvist et al. 2013), and N2a (Tremblay et al. 2010)). These are widely used in neurobiological research not only because their culture is quite simple, with unlimited proliferation potential, but also because they present some characteristics commonly found in neurons, including neurotransmitters, ion channels, receptors, and different neuron‐specific proteins (Barbosa et al. 2015). Additionally, most of these cell lines underwent genetic modifications to provide them with genetic homogeneity and stability over multiple passages, which offers the advantage of reducing biological variability and enhancing their reliability and reproducibility in experiments (Gordon et al. 2013). However, the intrinsic physiology of these cell lines differs significantly from that of primary neurons. To mitigate this, their culture conditions are often modified (such as through the addition of specific growth factors) to induce their differentiation towards a more neuron‐like phenotype (Ray et al. 2014). Nevertheless, despite their genetic consistency, these differentiation protocols can introduce phenotypic variability, as the effectiveness of the process may vary (Gordon et al. 2013).

Among the wide range of immortalized cell lines available for neurobiological research, human‐derived cell lines are particularly valuable as they closely replicate human gene and protein expression, including human‐specific isoforms of proteins relevant to neurogenesis, features that are absent in rodent primary cultures or rodent immortalized cell lines (Martin et al. 2022). Additionally, cells with a non‐neuronal origin have limited ability to replicate the molecular and functional characteristics of authentic neurons (Gordon et al. 2013). Taken together, human neuroblastoma cell lines currently represent the most suitable immortalized cell line for investigating the mechanisms underlying human neuronal function (Shastry et al. 2001). Accordingly, these models will be discussed in greater detail in the following sections.

#### 2.2.1 Neuroblastoma Cell Lines

Neuroblastoma‐derived cells present two distinct morphological phenotypes: S‐type, substrate‐adherent/epithelial‐like cells, and N‐type, neuroblast‐like cells (Voigt et al. 2000), which can either adhere or float in culture media (Şahin et al. 2021). N‐type cells are typically preferred for experiments, with some protocols recommending the use of only floating cells to obtain neuronal‐like phenotypes (Kovalevich and Langford 2013). This can be achieved by minimizing trypsin incubation time, which selectively detaches the more trypsin‐sensitive N‐type cells while leaving the epithelial‐like S‐type cells attached to the dish (Shipley et al. 2016). N‐type cells are typically favored for neuroscience research because they have the capacity to express a wide range of neuronal markers, which are absent in epithelial‐like cells (Korecka et al. 2013). Additionally, although at very low levels, these cells express cholinergic and glutamatergic markers even in their undifferentiated state (Filograna et al. 2015).

When treated with different differentiation‐inducing agents, their proliferation rate decreases, and they tend to adopt a morphology more similar to primary neurons. They extend long, thin, and branched cytoplasmic projections analogous to neurites, which are presumed to participate in the establishment of synaptic‐like connections, a key feature of neurons (Kaya et al. 2024). Moreover, supplementation of the culture media with specific differentiation factors promotes the expression of neuronal markers characteristic of the neuronal‐like phenotype adopted by these cell lines (Kovalevich and Langford 2013), as detailed in Table 1.

**TABLE 1:** Neuronal differentiation features of human neuroblastoma‐derived cell lines in 2D culture under different treatments.

Differentiation media	Morphological changes	Differentiation markers	Suggested differentiation pathway	Acquired neuronal‐like phenotype	References
*SH‐SY5Y cell line*					
▪ DMEM/F12 + 2% B27 ▪ DMEM/F12 + 5% B27 ▪ DMEM/F12 + 10 μM RA + 20 ng/mL BDNF Incubated for 5d	B27, RA, and BDNF‐treated conditions resulted in a pyramidal shaped cell body with longer projections	Upregulation of βIII‐tubulin in B27, RA, and BDNF‐treated cells	N/A	RA, BDNF and B27 treatments resulted in a glutamatergic phenotype	(Martin et al. 2022)
▪ DMEM/F12 + 10 μM RA + 20 ng/mL BDNF ▪ DMEM/F12 + 10 μM RA + 0.1% B27 ▪ DMEM/F12 + 10 μM RA + 2% B27 ▪ DMEM/F12 + 10 μM RA + 0.1% B27 + 20 ng/mL BDNF Incubated for 5d	B27 does not improve the effectiveness of RA and BDNF at promoting neurite extension	10 μM RA + 2% B27 treatment for 96 h decreased VMAT2, TH, and increased GLUL, VGLUT1, and GLS	N/A	RA, BDNF and B27 treatments resulted in a glutamatergic phenotype	(Martin et al. 2022)
▪ DMEM/F12 + 1% FBS + 10 μM RA ▪ DMEM/F12 + 1% FBS + 10 μM RA and 50 ng/mL BDNF supplementation at 4d Incubated for 7d	Extensive neurite outgrowth and branching	Upregulation of SYP, PSD‐95, ChAT, GLAST. Downregulation of TH. No changes in glutamatergic marker GLUL	N/A	Cholinergic with incomplete glutamatergic phenotype	(Targett et al. 2024)
▪ DMEM +15% FCS + 10 μM RA + 2 nM NGF ▪ DMEM +15% FCS + 10 μM RA + 2 nM BDNF ▪ DMEM +15% FCS + 10 μM RA + 2 nM NT‐3 ▪ DMEM +15% FCS + 10 μM RA + 2 nM NT‐4/5	Neurite outgrowth in cells treated with BDNF and NT‐4/5 (and to a lesser extent, NT‐3)	N/A	MAPK and PI3K/Akt pathway	N/A	(Encinas et al. 1999)
▪ MEM + 3% FBS + 2 mM L ‐glutamine +10 μM RA Incubated for 7d	Neurite outgrowth 24 h after the application of RA	Downregulation of Id1. Upregulation of NSE, SYP, SAP97, and NeuN. No alterations in NF, MAP2, DAT, and TH	Akt pathway	N/A	(Cheung et al. 2009)
▪ MEM/F12 supplemented with non‐essential aminoacids, L‐glutamine, and sodium pyruvate +0.2% FBS + 0.1 nmol/L PACAP‐38 ▪ MEM/F12 supplemented with non‐essential aminoacids, L‐glutamine, and sodium pyruvate +0.2% FBS + 0.1 to 1000 nmol/L VIP ▪ MEM/F12 supplemented with non‐essential aminoacids, L‐glutamine, and sodium pyruvate +0.2% FBS + 0.1 to 10 000 nmol/L Ro‐25‐1553 Incubated for 4d	≥ 10 nmol/L of PACAP‐38 induce neuritogenesis, while much higher concentrations of VIP (1000 nmol/L) are required. No statistical differences were seen with Ro 25–1553	≥ 1 nmol/L of PACAP‐38 increased the expression of ChAT but not TH	P38 MAP kinase pathway	N/A	(Monaghan et al. 2008)
▪ DMEM/F12 + 2 mM glutamine +1% FBS + 10 μM RA Incubated for 4, 7 and 10d	N/A	Upregulation of TH, NSE and NeuN in all timepoints, with a downregulation of Nestin at day 7	N/A	Dopaminergic phenotype	(Lopes et al. 2010)
▪ DMEM/F12 + 10% FBS + 15 nM TPA ▪ DMEM/F12 + 10% FBS + 10 μM RA ▪ DMEM/F12 + 10% FBS + 10 nM staurosporine Incubated for 7d	Neurite outgrowth was highest in staurosporine‐treated cells, followed by RA treatment, while TPA‐treated cells showed no change; The number of neurites per cell was not significantly altered by any treatment	RA and staurosporine but not TPA upregulated βIII‐tub and NF. TPA and RA did not affect the expression of TH, AADC, VMAT2, and DβH but slightly upregulated ChAT, AChE, VGLUT1, and GadD1. Staurosporine upregulated VMAT2 and DβH	N/A	Staurosporine induced a noradrenergic phenotype TPA and RA induced a loss of the catecholaminergic phenotype	(Filograna et al. 2015)
▪ Phase 1: DMEM +4 mM L ‐glutamine +5% FBS + 10 μM RA incubated for 3d and replaced by the media of phase 2 ▪ Phase 2: Neurobasal‐A + 200 nM L ‐glutamine +1% N‐2 supplement +50 ng/mL BDNF incubated for 3d	Increase in neurites' area	Upregulation of βIII‐tub and NF. Downregulation of SOX3 but upregulation of Nestin, at the end of phase 1. Downregulation of EN1, SOX2, and PAX6, but no difference of Nestin at the end of phase 2. No difference in NeuN expression. No difference in mRNA levels of TH and DAT	N/A	N/A	(Forster et al. 2016)
▪ DMEM +15% FCS + 10 μM RA incubated for 5d and 10d ▪ DMEM +15% FCS + 10 μM RA incubated for 5d followed by DMEM +50 ng/mL BDNF for 7d	N/A	Upregulation of NF‐L and NF‐M in untreated and RA‐only treated cells. Upregulation of NSE and in all treated conditions. Upregulation of GAP‐43 in all treated conditions, but returns to baseline levels after the first day of BDNF exposure	N/A	N/A	(Encinas et al. 2000)
▪ DMEM +10% FBS + 10 μM RA ▪ DMEM +10% FBS + 10 μM RA + 80 nM to 10 μM SST Incubated for 7d	SST (2 μM) enhanced neurite outgrowth with strong βIII‐tub co‐expression. SST knockdown reduced neurite outgrowth	RA + 10 μM SST increased MAP2 levels at day 3, which returned to baseline by day 7. RA + 2 and 10 μM SST treatments elevated Tau levels at day 7 compared to RA alone. βIII‐tub levels remained unchanged across all conditions	ERK 1/2 pathway	N/A	(Paik et al. 2019)
▪ DMEM/F12 + 1 mM glutamine + CGF ▪ DMEM/F12 + 1 mM glutamine +10 μM RA ▪ DMEM/F12 + 1 mM glutamine +10 μM RA + CGF Incubated for 3d	Neurite outgrowth in all tested conditions. Increase in NGF and BDNF released into the culture medium, in all conditions	Upregulation of NeuN, SYP, and βIII‐tubulin in all treated conditions, except in RA‐only treated conditions	N/A	N/A	(Borsani et al. 2020)
▪ DMEM +3 to 5% FBS + 10 μM RA incubated for 6d ▪ DMEM +3 to 5% FBS + 10 μM RA incubated for 6d and replaced with neurobasal +2 mM glutamine +1% N2 + 50 ng/mL BDNF incubated for additional 6d	Neurite outgrowth in BDNF‐treated condition with established synaptic connections	BDNF‐treated condition showed increased Tau, MAP2, PSD‐95, SHANK3, and SYP expression	N/A	N/A	(Hromadkova et al. 2020)
▪ DMEM (low glucose 0.9 g/L) + 0.876 g/L glutamine +1.19 g/L HEPES +3.7 g/L sodium bicarbonate +0.11 g/L sodium pyruvate +1% FBS + 10 μM RA Incubated for 3d	Neurite outgrowth and cellular area expansion	No alterations in NSE, MAP2, and βIII‐tubulin gene expression. Upregulation of the immunocontent of NeuN, MAP2, and βIII‐tubulin. Upregulation of the immunocontent of TH	N/A	N/A	(Simões et al. 2021)
▪ DMEM/F12 + 1% FBS + 10 μM RA ▪ DMEM/F12 + 1% FBS + 10 μM RA + 50 ng/mL BDNF Incubated for 7d	Longer and more branched neurites in RA + BDNF treated cells	Upregulation of CHRNA6, CHRM4, CHRM3, CHRNA4, BACE2, Tau, ADAM10, and SCL18A in RA + BDNF treated cells. Upregulation of AChE in both conditions. Upregulation of vAChT, CDK5, and PSEN1 in both conditions. Upregulation of DAT in RA‐only treated cells	N/A	Cholinergic phenotype in RA + BDNF treated cells. Dopaminergic phenotype in RA + only treated cells	(de Medeiros et al. 2019)
▪ DMEM +10% FBS + 0.01 μM non‐essential aminoacids +10 μM RA ▪ DMEM +10% FBS + 0.01 μM non‐essential aminoacids +80 nM TPA ▪ DMEM +10% FBS + 0.01 μM non‐essential aminoacids +80 nM TPA + 10 μM RA Incubated for 6d	N/A	Upregulation of TH, D2 and D3 receptors, DAT and VMAT in RA + TPA treated cells. RA‐only treated cells had no effect on the levels of TH, DAT, and D3 receptor. Upregulation of TH and DAT in TPA‐only and RA + TPA treated cells but downregulation in VMAT. All conditions increased the levels of MAP2 and β‐Actin. RA + TPA treated cells presented high DA uptake, sensitivity to MPP+ toxicity, which was inhibited by a DAT blocker	N/A	Dopaminergic phenotype in RA + TPA treated cells	(Presgraves et al. 2003)
▪ Phase 1: EMEM +2.5% FBS + 2 mM glutamine +10 μM RA incubated for 7d and replaced by the media of phase 2 ▪ Phase 2: EMEM +1% FBS + 2 mM glutamine +10 μM RA incubated for 3d and replaced by the media of phase 3 ▪ Phase 3: Neurobasal +1× B27 + 20 mM KCl + 2 mM Glutamaxl +50 ng/mL BDNF +2 mM cAMP +10 μM RA incubated for 7d	Extensive and elongated neuritic projections	Fully differentiated cells are immunopositive for SMI31, and MAP2	N/A	N/A	(Shipley et al. 2016)
▪ DMEM +10% FBS + 8 μM RA Incubated for 5d	Neurite outgrowth	Upregulation of CFL1, BASP1, neuromodulin, MAP1B, and MAP2. Downregulation of. KIF11. Downregulation of MCM 2–7 complex proteins, LIG1, FEN1, RFC4, RFC5, PCNA, PFDN6, HSPA14, the eight members of TRiC/CCT, HSPD1, HSPA9, and HSPE1, TRAP1	N/A	N/A	(Leung et al. 2024)
*IMR‐32 cell line*					
▪ EMEM +10% FBS + 8 μM RA Incubated for 5d	Neurite outgrowth	Upregulation of CFL1, BASP1, neuromodulin, MAP1B, MAP2, and downregulation of KIF11. Downregulation of MCM 2–7 complex proteins except MCM5, LIG1, FEN1, RFC4, RFC5, and PCNA	N/A	N/A	(Leung et al. 2024)
▪ DMEM +2% FBS + 10 μM RA ▪ DMEM +2% FBS + 50 μM IS00384 ▪ DMEM +2% FBS + 50 μM sildenafil Incubated for 4d	Neurite outgrowth in all tested conditions	All conditions were immunopositive for βIII‐tubulin, NeuN, and NFH. Increase in p‐C3G/C3G, p‐AMPK/AMPK, and pACC/ACC levels in all conditions, with the maximum effect showed by IS00384	AMPK‐ACC and PI3K‐Akt pathways	N/A	(Dar et al. 2020)
▪ DMEM +10% FBS + 10 μM RA Incubated for 7d	Neurite outgrowth	Upregulation of BDNF, NGF, NSE, TH, and βIII‐tubulin	JAK/STAT pathway	N/A	(Kotapalli et al. 2017)
▪ DMEM +10% FBS + 1× Glutamax +0.7 μM CDDO ▪ DMEM +10% FBS + 1× Glutamax +10 μM RA ▪ DMEM +10% FBS + 1× Glutamax +10 μM RA + 0.7 μM CDDO Incubated for 5d	Neurite outgrowth in all tested conditions, but RA + CDDO had the highest neurite number and length	Downregulation of βIII‐tubulin in all conditions by day 3, but upregulation in the RA + CDDO group by day 5. Upregulation of NSE in all conditions by day 3, but slight decrease by day 5. Upregulation of NSE in CDDO treated conditions	RA activated ERK1/2‐CREB pathway. CDDO inhibited. CREB activation via active PPARg signaling	N/A	(Chaudhari et al. 2017)
▪ DMEM +10% FCS + 100 mM Glutamax +4 μM RA ▪ DMEM +10% FCS + 100 mM Glutamax +30 μM fatty acids (ALNA, DHA, LA, AA, and OA) or 100 μM CFA incubated for 3d	Short neurite formation in cells treated with RA, ALNA, DHA, CFA, and OA but extensive neurite formation with LA and AA	N/A	Fatty acids activate the PPAR pathway	N/A	(Burdge et al. 2001)

Abbreviations: AA, arachidonate; AADC, aromatic L‐amino acid decarboxylase; AchE, acetylcholinesterase; ADAM10, a disintegrin and metalloproteinase domain‐containing protein 10; AKT, protein kinase B; ALNA, α‐linolenate; AMPK‐ACC, AMP‐activated protein kinase–acetyl‐CoA carboxylase axis; BACE2, beta‐site APP cleaving enzyme 2; BASF1, brain abundant membrane‐attached signal protein 1; BDNF, brain‐derived neurotrophic factor; C3G/p‐C3G, guanine nucleotide exchange factor/p–phosphorylated form; cAMP, cyclic adenosine monophosphate; CDDO, 2‐cyano‐3,12‐dioxooleana‐1,9 (11)‐dien‐28‐oic acid (partial PPARγ agonist); CDK5, cyclin‐dependent kinase 5; CFA, clofibric acid; CFL1, cofilin 1; CGF, concentrated growth factors; ChAT, choline acetyltransferase; CHRM3, muscarinic acetylcholine receptor M3; CHRM4, muscarinic acetylcholine receptor M4; CHRNA4, cholinergic receptor nicotinic alpha 4 subunit; CHRNA6, cholinergic receptor nicotinic alpha 6 subunit; DAT, dopamine transporter; DHA, docosahexaenoate; DMEM, Dulbecco's Modified Eagle Medium; DβH, dopamine beta‐hydroxylase; EMEM, Eagle's Minimum Essential Medium; EN1, engrailed homeobox 1; EPA, eicosapentaenoate; ERK1/2, extracellular signal‐regulated kinases 1 and 2; F12, Ham's F12 nutrient mixture; FBS, fetal bovine serum; FCS, fetal calf serum; FEN1, flap endonuclease 1; GAD1, glutamate decarboxylase 1; GAP‐43, growth associated protein 43; GLAST, glutamate aspartate transporter; GLS, glutaminase; GLUL, glutamine synthetase; HEPES, 4‐(2‐hydroxyethyl)‐1‐piperazineethanesulfonic acid (buffering agent); HSPA14, heat shock protein family A (Hsp70) member 14; HSPA9, heat shock protein family A (Hsp70) member 9; HSPD1, heat shock protein family D member 1; HSPE1, heat shock protein family E member 1; JAK/STAT, Janus kinase/signal transducer and activator of transcription pathway; KIF11, kinesin family member 11; LA, linoleate; LIG1, DNA ligase I; MAP1B, microtubule‐associated protein 1B; MAP2, microtubule‐associated protein 2; MAPK, mitogen‐activated protein kinase; MCM 2–7, minichromosome maintenance complex proteins 2 to 7; MCM5, minichromosome maintenance complex component 5; MEM, Minimum Essential Medium; mRNA, messenger RNA; N/A, not applicable; NeuN, neuronal nuclei; NF, neurofilament; NFH, neurofilament heavy chain; NF‐L, neurofilament light chain; NF‐M, neurofilament medium chain; NGF, nerve growth factor; NSE, neuron‐specific enolase; NT‐3, neurotrophin‐3; NT‐4/5, neurotrophin‐4/5; OA, oleate; PACAP‐38, pituitary adenylate cyclase‐activating polypeptide‐38; pACC/ACC, phosphorylated acetyl‐CoA carboxylase/total ACC; pAMPK, phosphorylated AMP‐activated protein kinase; PAX6, paired box 6; PCNA, proliferating cell nuclear antigen; PFDN6, prefoldin subunit 6; PI3K, phosphoinositide 3‐kinase; PSD‐95, postsynaptic density protein 95; PSEN1, presenilin 1; RA, retinoic acid; RFC4, replication factor C subunit 4; RFC5, replication factor C subunit 5; RO‐25‐1553, selective VPAC2 receptor agonist; SAP97, synapse‐associated protein 97; SCL18A, solute carrier family 18; SHANK3, SH3 and multiple ankyrin repeat domains protein 3; SMI31, antibody marker for phosphorylated neurofilaments; SOX2, SRY‐box transcription factor 2; SOX3, SRY‐box transcription factor 3; SST, somatostatin; SYP, synaptophysin; Tau, microtubule‐associated protein tau; TH, tyrosine hydroxylase; TPA, 12‐O‐tetradecanoylphorbol‐13‐acetate; TRAP1, TNF receptor‐associated protein 1; TRiC/CCT, TCP‐1 Ring Complex/Chaperonin Containing TCP1; vAChT, vesicular acetylcholine transporter; VGLUT1, vesicular glutamate transporter 1; VIP, vasoactive intestinal peptide; VMAT2, vesicular monoamine transporter 2.

In this work, the SH‐SY5Y and IMR‐32 cell lines were explored in greater detail, as they are among the most commonly used human neuroblastoma models for studying neuronal differentiation (Leung et al. 2024). Indeed, the SH‐SY5Y human neuroblastoma cell line is one of the most used cell models in neuroscience (Kaya et al. 2024; Campos Cogo et al. 2020). It is a thrice‐cloned subline from the parental SK‐N‐SH cell line, originally derived from a metastatic bone tumor biopsy of a patient diagnosed with neuroblastoma of sympathetic adrenergic ganglial origin. In their undifferentiated state, SH‐SY5Y cells present a polygonal soma with few short processes (Alaylıoğlu et al. 2023) and express neuronal markers associated with proliferation and immaturity (Martin et al. 2022). This indicates that in this state, they may be more suitable as a model for neural progenitor cells (some reports mention they resemble immature catecholaminergic neurons (Kovalevich and Langford 2013)). However, differentiation is required to accurately replicate the molecular processes and functions of mature neurons (Martin et al. 2022).

When treated with differentiation‐inducing agents like retinoic acid (RA), saurosporine, BDNF, nerve growth factor (NGF), or other less commonly used compounds, these cells can be driven to differentiate into various neuronal phenotypes (Kovalevich and Langford 2013). However, there is no standardized protocol for this process and according to some reports, different differentiation agents lead to a range of neuronal phenotypes, including cholinergic, adrenergic, noradrenergic, dopaminergic (Langerscheidt et al. 2024), or glutamatergic (Martin et al. 2022) neurons.

Analysis of Table 1 reveals that different differentiation protocols can have varying effects on the neuronal profiles of the human neuroblastoma cell lines SH‐SY5Y and IMR‐32 (Forster et al. 2016). Some protocols supplement culture media with only RA, since this vitamin A derivative has been shown to induce neuronal differentiation through multiple mechanisms (Shipley et al. 2016). The primary mechanism of RA action involves binding to and activating retinoic acid receptors (RARs), which trigger a wide range of cellular responses. These include upregulation of pro‐neuronal genes such as neural cell adhesion molecule 2 (NCAM2), TrkB, and Netrin G2 (NTNG2), suppression of proliferative pathways and non‐neuronal genes like N‐myc proto‐oncogene (MYCN), bone morphogenetic protein 7 (BMP7), and Achaete‐scute homolog 1 (ASCL1), and activation of signaling cascades that increase the expression of other neuronal differentiation markers and structural proteins essential for neurite outgrowth (Korecka et al. 2013). However, most differentiation protocols combine RA with BDNF, as RA enhances neuronal differentiation potential by upregulating the aforementioned TrkB receptor, thereby increasing the cells' responsiveness to neurotrophins like BDNF and leading to more pronounced neuronal maturation (Dravid et al. 2021). These results are confirmed in the study of Medeiros et al. who reported that supplementing the media with RA prior to a BDNF treatment was crucial for promoting the formation of longer, more branched neurites and a robust neuritic network, when compared to a RA‐only treatment (de Medeiros et al. 2019). They confirmed that this effect was mediated by TrkB activation, which was enabled by the prior RA treatment, allowing BDNF to drive morphological differentiation. Indeed, it has been reported that RA treatment alone typically yields cells with immature synaptic properties and mixed phenotypes, as only ~20% of cells fully adopt neuronal traits (Alaylıoğlu et al. 2023; Yang et al. 2016), and prolonged exposure to this compound leads to an increase of S‐type cells, as these cells are resistant to the growth inhibitory effects of RA (Encinas et al. 2000). This approach is more suitable for studying early differentiation mechanisms, where full maturation isn't required. For functional synaptic studies or disease modeling requiring mature neurons, sequential RA + BDNF remains superior (Alaylıoğlu et al. 2023). Therefore, most protocols rely on the use of RA and BDNF, combined with serum deprivation, which helps favor neuronal differentiation over proliferation (Howard et al. 1993).

Other studies have evaluated alternative differentiation agents by comparing their effects not only to untreated controls but also to cells treated with RA alone, which remains the most widely used and established method for inducing neuronal differentiation in SH‐SY5Y cells. For instance, Borsani et al. investigated the efficacy of a growth factor cocktail both on its own and in combination with RA, using RA‐only treatment as a reference. They found that while RA alone could initiate neurite outgrowth, it was insufficient to drive full neuronal maturation, as indicated by the low expression levels of mature neuronal markers such as NeuN and synaptophysin (Borsani et al. 2020). However, a key limitation of this study lies in the short duration of the differentiation protocol, only 3 days. While this timeframe may be enough to observe early signs of differentiation, such as neurite initiation, it likely does not give cells enough time to reach full neuronal maturation (Shipley et al. 2016). Therefore, the observed low expression of mature neuronal markers may not reflect the limitations of RA, but instead an incomplete timeline for full differentiation to occur. Having said this, longer‐term studies are often required to determine the real impact of treatments on the differentiation of these cells (Strother et al. 2021).

Few differentiation protocols use NGF as a differentiation agent, likely because the response of SH‐SY5Y cells to this neurotrophin is highly variable. This variability depends on factors such as cell passage number, prior treatment conditions, and notably, baseline TrkA expression (the high‐affinity receptor required for NGF signaling) (Poluha 1992; Jensen et al. 1992). These findings were confirmed by Encinas et al., who reported that the addition of 2 nM NGF to SH‐SY5Y cultures resulted in little to no neuritic outgrowth, comparable to cultures lacking any neurotrophin treatment (Encinas et al. 1999).

Besides conventional differentiation agents, other compounds have been explored for their potential to induce differentiation in SH‐SY5Y cells. For example, Monaghan et al. treated SH‐SY5Y cells with the neuropeptide pituitary adenylate cyclase‐activating polypeptide‐38 (PACAP‐38) and observed induction of neuritogenesis. This effect was attributed to PACAP‐38's binding to PAC_1_ receptors, which increases intracellular cyclic adenosine monophosphate (cAMP) levels and activates downstream signaling pathways, such as the extracellular signal‐regulated kinase (ERK) and p38 mitogen‐activated protein kinase (p38 MAPK) pathways, which promote neurite outgrowth and regulate gene expression required for neuronal maturation (Monaghan et al. 2008). Another study compared the effects of staurosporine and the phorbol ester 12‐O‐tetradecanoylphorbol‐13‐acetate (TPA) on neuronal differentiation and found that protein kinase C (PKC) may be involved in this process because staurosporine, a potent PKC inhibitor, contributed to a higher neurite outgrowth compared to TPA, which is a PKC activator (Filograna et al. 2015).

Additionally, although B27‐incubated SH‐SY5Y cells cannot be subcultured, they display reduced proliferation, longer processes, and a more differentiated, pyramidal‐like morphology compared to FBS‐treated cells. When compared to conventional differentiation protocols (10 μM RA and 20 ng/mL BDNF), B27 and RA + BDNF treatments both increased neuronal differentiation markers, consistent with previous findings that serum starvation promotes SH‐SY5Y differentiation. While B27 promotes neurite outgrowth, its combination with RA and BDNF does not further enhance neurite extension, suggesting that RA and BDNF alone are sufficient. By assessing different neuronal markers, the authors also concluded that BDNF drives SH‐SY5Y cells towards a more glutamatergic‐like phenotype (Martin et al. 2022).

But besides the differentiation agents used, several authors emphasize that the duration of the protocol also plays a critical role in determining the phenotype and maturity of these cells (Dravid et al. 2021). While most protocols use 7 days to differentiate SH‐SY5Y cells, several studies have shown that such short durations typically yield only partially differentiated cells that do not fully capture mature neuronal characteristics (Dravid et al. 2021). To better mimic fully mature neurons with extensive neurite outgrowth and functional properties, some studies suggest the use of longer differentiation protocols, which have been shown to produce terminally differentiated, neuron‐like cells, as described in (Encinas et al. 2000; Dravid et al. 2021). And although some studies may indicate that SH‐SY5Y cells can express mature neuronal markers, electrophysiological studies have shown that these cells lack the complex electrical activity characteristic of mature neurons, indicating that these cells do not achieve complete functional maturation (D'Aloia et al. 2024). While this is an extremely versatile cell line offering multiple advantages, these are highly sensitive cells that proliferate more slowly than other immortalized cell lines, making their culture and use in routine laboratory experiments quite challenging (Kaya et al. 2024).

One last important consideration when differentiating SH‐SY5Y cells is the passage number, which many protocols do not specify. Most studies reported in Table 1 use early passage cells (≤ 20) (Martin et al. 2022; Filograna et al. 2015; Targett et al. 2024; Monaghan et al. 2008; Paik et al. 2019; Borsani et al. 2020), while others use cells at higher passages (Forster et al. 2016; Simões et al. 2021). These cells may not show classic senescence signs, but their biochemical properties are significantly influenced by passage number, and it is recommended to keep it below 20 in all experiments to ensure consistency and reliability (Gómez‐Ramos et al. 2008).

IMR‐32 cells also retain the ability to differentiate into neuron‐like cells and share many characteristics with SH‐SY5Y cells. However, reportedly, they have a less diverse neurotransmitter profile, and they show less prolonged neurite outgrowth and undergo less morphological transformations when differentiated, reason why they are less commonly used for mature neuronal models (Leung et al. 2024). In a comparative proteomic study on the effects of RA on SH‐SY5Y and IMR‐32 cell lines, the molecular changes of these cells during differentiation were examined. While both lines showed broadly similar proteomic responses, some differences emerged. For example, molecular chaperones like prefoldin subunit 6 (PFDN6) and heat shock protein family A member 14 (HSPA14) were downregulated in SH‐SY5Y cells but upregulated in IMR‐32 cells. This contrast may suggest that SH‐SY5Y cells undergo a more efficient or less stressed differentiation process, making them more appropriate for studying normal neuronal brain processes, such as neurogenesis. In contrast, the increased chaperone expression in IMR‐32 cells may reflect higher proteotoxic stress, potentially limiting how accurately this model reflects physiological neuronal behavior (Leung et al. 2024).

Unlike SH‐SY5Y cells, IMR‐32 cells present some resistance to RA‐induced differentiation (Chaudhari et al. 2017), which could be attributed to their amplified MYCN gene that interferes with RA action through several suggested mechanisms, such as by repressing RA‐responsive genes, sustaining cell cycle progression, and enhancing RA degradation (Chlapek et al. 2018; Bell et al. 2007). This resistance was also confirmed by Burdge et al., who also found that certain fatty acids (although at higher concentrations than RA) promoted more extensive neurite outgrowth than RA (Burdge et al. 2001). A different study also reported that RA treatment in these cells induces neuronal differentiation pathways only when tumor protein D52 (TPD52) is expressed, suggesting that RA alone is insufficient to promote these differentiation‐related changes (Kotapalli et al. 2017). Furthermore, alternative compounds such as phosphodiesterase type 5 (PDE5) inhibitors (sildenafil and IS00384) have been shown to promote IMR‐32 differentiation by activating signaling pathways (specifically AMPK and PI3K/Akt) that overlap with those triggered by RA, which offers a potential alternative to overcome the RA differentiation resistance observed in MYCN‐amplified IMR‐32 cells (Dar et al. 2020).

In sum, SH‐SY5Y and IMR‐32 cells are both valuable human neuroblastoma cell models for studying neuronal differentiation. SH‐SY5Y cells exhibit robust differentiation potential, especially when treated with combined agents like RA and BDNF, but their full functional maturation remains challenging. In contrast, IMR‐32 cells demonstrate partial resistance to RA‐induced differentiation and rely on other compounds for effective differentiation. Understanding how variations in protocols influence the differentiation of these cells into different neuronal phenotypes is essential, and ultimately, the choice of differentiation method should be based on the desired neuronal phenotype.

#### 2.2.2 Culture Configurations

Recognizing that differentiated neuroblastoma cell lines often fail to fully replicate the morphological, biochemical, and functional characteristics of mature neurons, researchers have explored different strategies to enhance differentiation outcomes. One promising approach involves culturing these cells in more complex neurobiological models, such as 3D systems that better replicate the neural environment, as previously discussed for primary brain cell cultures (D'Aloia et al. 2024). For this purpose, SH‐SY5Y appears to be the only neuroblastoma cell line used for 3D modeling of the neural environment (Fabbri et al. 2023).

In a recent study, researchers have shown that a Growth Factor Reduced (GFR)‐based matrix helped SH‐SY5Y cells acquire features of electrically active cholinergic neurons, being able to establish functional networks with active synaptic structures and vesicles with directional sorting trafficking (D'Aloia et al. 2024). Another study took advantage of the nano‐sized fibrils that are extracellularly secreted by the bacterium *Gluconacetobacter xylinus* and modified its surface with collagen I to mimic the properties of the extracellular matrix. Electrophysiological recordings of SH‐SY5Y cells encapsulated in this matrix demonstrated functional action potential, which the authors attributed to enhanced cell–cell and cell‐matrix interactions. These interactions were thought to increase the cells' sensitivity to differentiation signals from both the surrounding media as well as from adjacent cells (Innala et al. 2014).

Besides improving the maturation of these cells, 3D cultures may also improve the efficiency of the differentiation towards a desired neuronal subtype. This is highlighted by the research conducted by Fiore et al., which demonstrated that a 3D culture system composed of silk‐hydrogel composite promoted the development of dopamine neuron markers while simultaneously decreasing the expression of markers for other neuronal subtypes, in contrast to the monolayer system (Fiore et al. 2022).

As in primary cultures, the co‐culture of neuroblastoma cells with other cell types, either in 2D or 3D conditions, is another viable alternative to improve their differentiation potential and enhance cell survival, as well as neuronal characteristics. For example, co‐culture of SH‐SY5Y cells with pre‐adipose PA6 cells and RA promoted their differentiation towards a more mature phenotype of motor neurons, since this stromal cell line can produce sonic hedgehog, a signaling molecule that plays a crucial role in promoting ventral neuronal identity. Authors from this study additionally reported a reduction in the time required to achieve a mature neuronal phenotype (Ferguson and Subramanian 2016), which is a factor that should also be addressed in other co‐culture studies.

### 2.3 Pluripotent Stem Cells

Similar to immortalized cells, stem cells can be differentiated into specific neuronal subtypes in a controlled manner and, due to their pluripotency and self‐renewal, can also be cultured long‐term before being differentiated (Bradford 2015). Being pluripotent, both embryonic stem cells (ESCs) and induced pluripotent stem cells (iPSCs) can be converted into different neuronal subtypes through a multi‐step process in which these cells are exposed to growth factors, inhibitors, and/or stimulators of different pathways involved in cell fate choices (Compagnucci et al. 2014).

ESCs have been the primary source of pluripotent stem cells used in research. These cells are derived from the inner cell mass of developing blastocysts, typically from leftover human embryos created during in vitro fertilization procedures, or alternatively, from rodent blastocysts (Ginis et al. 2004). Their source raises ethical concerns: human ESCs require the destruction of embryonic material, which has given rise to moral and political debates and led to strict ethical and regulatory control. In contrast, ESCs derived from nonhuman animal embryos, while still involving the use of embryos and the sacrifice of the donor mother animal, are generally viewed as more ethically acceptable, although their use also raises ethical concerns (Sugarman 2008), as previously discussed in Section 2.1.

ESCs attractiveness largely stems from their pluripotency, which allows them to self‐renew and differentiate into nearly all cell types in the body, including those that make up the three embryonic germ layers. This versatility makes these cells particularly valuable for replicating the diverse structures and developmental pathways that contribute to human development, including the formation and organization of neuronal structures (Evangelisti et al. 2024). Despite their usefulness, particularly in developmental studies, efficiently converting ESCs into specific cell types in vitro has remained their major limitation. Various protocols have been developed to guide cell fate decisions to generate neural progenitors, followed by additional steps to generate specific neuronal subtypes. But these protocols are often complex, expensive, and labor‐intensive, often demanding weeks of specialized culture conditions to generate cells capable of differentiating into neurons (Li et al. 2009).

IPSCs emerged as an alternative to ESCs not only because they are ethically sourced, but also because they hold significant promise in regenerative medicine, since they can be generated from a patient's own somatic cells, reprogrammed into a pluripotent state, and then differentiated into target neurons for transplantation, without the risk of immune rejection (Hu et al. 2010). Their reprogramming can be generated through the viral delivery of the Yamanaka factors (pluripotency‐associated transcription factors), but because this protocol requires specialized knowledge, equipment, and reagents (Al Abbar et al. 2020), ready‐to‐use iPSC lines are readily available for purchase from several commercial providers. In contrast, although numerous approved lines of human ESCs exist, with the National Institutes of Health (NIH) maintaining a registry that provides information on them, their availability is more limited and dependent on usage restrictions (National Institutes of Health, n.d.).

Once reprogrammed and starting with a stem cell state, under proper conditions, both ESCs and iPSCs can progress through stages characterized by NSC and neural progenitor cell traits, followed by an immature neuron phase, ultimately reaching a more mature state (Bradford 2015). Although iPSCs‐derived neurons can develop electrophysiological properties, their ability to achieve the functional maturity of native neurons remains controversial (Mateos‐Aparicio et al. 2020). Reprogramming inherently resets cellular age to an embryonic‐like state, erasing aging markers present in adult neurons (Cornacchia and Studer 2017). Furthermore, most protocols rely on short‐term cultures (Mateos‐Aparicio et al. 2020), which exacerbates this limitation, hindering their utility for modeling late‐onset neurological disorders, such as Alzheimer's disease (Cornacchia and Studer 2017). While there are no definite criteria to classify neurons as functionally mature, features of immaturity commonly reported include depolarized resting membrane potentials, higher input resistance, smaller membrane capacitance, slower and lower amplitude action potentials, and less developed synaptic activity, when compared to primary adult neurons (Mateos‐Aparicio et al. 2020). To mitigate these issues, several strategies can be implemented to promote further maturation, including prolonged culture times, co‐culture with other cells (such as astrocytes), the use of artificial extracellular matrices to recapitulate certain aspects of tissue architecture, and even transplantation into an in vivo environment such as a mouse brain, where neurons can follow the host developmental program and further mature (Mateos‐Aparicio et al. 2020; Cerneckis et al. 2024).

Despite these challenges in achieving full maturity, the pluripotent origin of iPSCs‐derived neurons provides a distinct advantage: unlike primary cultures and immortalized cells, where the transition between undifferentiated and differentiated states is often unclear, with these cells, researchers have precise control over the differentiation process and can interrupt neurogenesis at specific milestones, which allows detailed analysis of each intermediate stage (Compagnucci et al. 2014). The factors regulating the neural induction of pluripotent cells are well understood, but their precise control in vitro is another challenge. Overall, neuronal fate commitment and differentiation in these cells can be achieved through a direct genetic differentiation method, which is achieved by delivering cDNA encoding a neuronal gene into the cell, where it will act by repressing or activating specific target genes, or by an indirect chemical differentiation method where the cell culture is enriched with pro‐neuronal growth factors and pharmacological agents that act on receptors, enzymes, and transcription factors to block a non‐neuronal fate (Telias 2023). Currently, there is a variety of protocols available, which often leads to inconsistent differentiation conditions, resulting in a heterogeneous neuronal population at various stages of maturation, which further complicates the replication of results. For this reason, it is crucial to functionally characterize the derived neurons to confirm their identity and functionality. Techniques such as electrophysiological recordings, morphological characterization with neuronal markers, and gene expression analysis are often employed to assess the maturation state and functionality of iPSC‐derived neurons (Bradford 2015). In this context, Compagnucci et al. provide a comprehensive overview of different protocols and outcomes involved in differentiating pluripotent stem cells into various neuronal subtypes, which not only guides researchers in generating specific neuronal subtypes but may also be useful for those aiming to standardize and optimize their differentiation strategies and to identify possible challenges in the process (Compagnucci et al. 2014).

#### 2.3.1 Culture Configurations

It can be difficult to make an all‐encompassing statement about the protocols currently used for studying neurogenesis from pluripotent stem cells, as they vary significantly in terms of strategy and efficiency. Some of the most common approaches include embryoid body (EB) formation, adherent monolayers for rosette formation, spheroids, hydrogels, stromal feeder layers, and organoids. Among these, EB‐based, monolayer‐derived neural rosettes, and organoids have been the most widely used (Slanzi et al. 2020).

Protocols for inducing EB rely on culturing pluripotent stem cells in suspension in neural induction medium, allowing them to spontaneously aggregate into spherical structures (Rungarunlert 2009). Different protocols allow EBs to grow for different periods of time, but once established, EBs mimic early embryonic development by giving rise to cells from all three germ layers (ectoderm, mesoderm, and endoderm) (Dhara and Stice 2008). Of note, EBs do not inherently model neurogenesis but broadly simulate early stages of embryogenesis and require specific cues to induce neuroectodermal fate (Liyang et al. 2014). Since only ectoderm‐derived cells give rise to neurons, it is critical to promote neuroectodermal lineage commitment within these cultures (Slanzi et al. 2020). A widely used method to transform pluripotent stem cells into neurons through EB formation is the 4−/4+ protocol. In this strategy, EBs are allowed to form and spontaneously differentiate for four days (4−), followed by four days (4+) of treatment with RA to induce neural differentiation. One of its main drawbacks is the fact that it gives rise to a mix of neuronal subtypes (Liyang et al. 2014). Another technical challenge in EB‐based systems is controlling their size and uniformity, as larger aggregates can lead to necrotic centers and smaller EBs do not survive during differentiation (Pettinato et al. 2014). Additionally, EB fusion (where multiple EBs merge into one) exacerbates these issues by increasing core necrosis (Rungarunlert 2009). To address this, researchers have employed methods such as hanging drop techniques (Tasoglu and Demirci 2013), or micro‐well arrays (Hwang et al. 2009) to create size‐controlled EBs.

Recently, pluripotent cells have been plated in a single layer on a coated surface (typically using substrates like Matrigel, collagen, fibronectin, or laminin) or with feeder cells to achieve a monolayer culture system (Dhara and Stice 2008). In this simpler system, cells are cultured in defined serum‐free conditions, with added supplements (generally RA) and inhibitors of BMPs (such as noggin), a group of growth factors known to block neural development (Abranches et al. 2009). If a feeder layer is present, this is typically removed around day 10. At this time, cells are already committed to neuronal lineages and present neural rosettes (radially arranged, neuroepithelial‐like structures that resemble the developing neural tube) in culture. These rosettes can be manually isolated and expanded to enrich for neuronal progenitors (Dhara and Stice 2008). These progenitors can then be further differentiated into specific neuronal subtypes using targeted cues such as fibroblast growth factors (FGFs), Wnt, or other small molecules, depending on the desired lineage, as reviewed in (Tao and Zhang 2016). In contrast to EB, which is a more general model of early embryonic development that requires neural‐inducing cues (Liyang et al. 2014), neural rosettes represent a more advanced stage, reflecting a committed neural fate that mimics the early neural tube and the emergence of neural progenitor cells (Elkabetz et al. 2008) (Table 2).

**TABLE 2:** Comparison of pluripotent stem cell culture systems for neurogenesis studies.

Culture system	Self‐organization	Spatial complexity	Key advantages	Limitations
Neural rosette	Yes (radially organized), 2D (Miotto et al. 2023)	Moderate (neural‐specific) (Miotto et al. 2023)	Highly accessible (Miotto et al. 2023)	Lacks 3D architecture and in vivo like patterning (Adlakha 2023), hard scalability (Frazier et al. 2025), variability (Townshend et al. 2020)
Embryoid Bodies	Yes (basic and unpatterned), 3D (ten Berge et al. 2008)	Moderate (multi‐lineage) (Dhara and Stice 2008)	Scalable (Sato et al. 2016)	Low neural specificity (Erceg et al. 2008), variable size with risk of necrosis (Pettinato et al. 2014)
Organoid	Yes (highly patterned), 3D (Zhao and Haddad 2024)	High (region‐specific, layered) (Jacob et al. 2021)	Physiologically relevant (Kim and Chang 2023)	High variability, risk of necrosis, technical demand (Urrestizala‐Arenaza et al. 2024)

**FIGURE 2:**
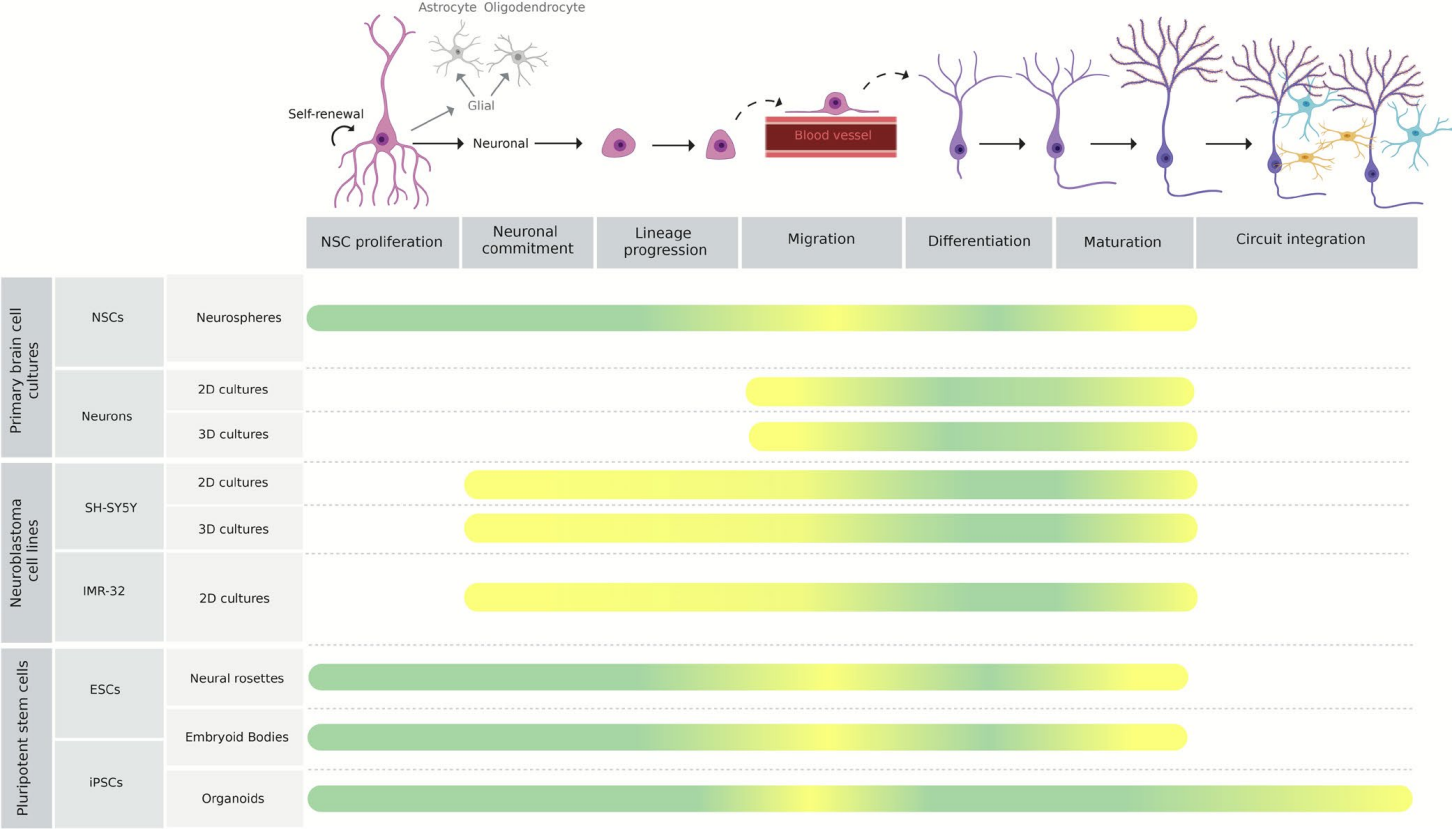
Simplified heatmap comparing the ability of different in vitro cell models to recapitulate sequential stages of neurogenesis. The figure illustrates how primary NSC‐derived neurospheres, primary neurons in 2D and 3D formats, immortalized neuroblastoma cell lines (SH‐SY5Y and IMR‐32) also in 2D and 3D formats, and pluripotent stem cell (ESC/iPSC)‐derived systems (neural rosettes, embryoid bodies, and organoids) reproduce distinct phases of neurogenesis. Early events such as NSC proliferation, neuronal commitment, and lineage progression are best modeled by stem cells (NSCs, ESCs, and iPSCs), as these cells naturally undergo self‐renewal and fate specification processes that precede neuronal differentiation (Tang et al. 2017; Ciceri et al. 2024) and cannot be recapitulated by post‐mitotic neuronal models (Bonnefont and Vanderhaeghen 2021). To some extent, neuroblastoma cell lines can also mimic these stages, as some protocols induce cell cycle exit while promoting the acquisition of neuronal characteristics, modeling the transition from a dividing precursor to a post‐mitotic neuron (Kovalevich and Langford 2013). Differentiation is captured across all cell types and configurations (Sahu et al. 2019; Kaya et al. 2024; Chaudhari et al. 2017; Liyang et al. 2014; Elkabetz et al. 2008; Park et al. 2023; Theocharatos et al. 2013), while neuronal maturation is best captured in brain organoids (Azari and Reynolds 2016; Sun et al. 2022). Neuronal migration, less frequently modeled in vitro, remains technically challenging to reproduce in 2D cultures (Azzarelli et al. 2017). Although most models can mimic certain molecular and cytoskeletal aspects of migration, this process is best captured by brain organoids due to their higher architectural complexity (Aili et al. 2024). Color coding reflects fidelity to each developmental stage. The classification is qualitative and interpretative rather than quantitative. It was derived from how extensively each stage is described in the literature for a given model and from the degree to which hallmark features of that stage have been experimentally demonstrated. Green (well represented): The model is commonly used for studying this stage of neurogenesis and is reported to recapitulate its key features. Yellow (partially represented): Some hallmark features of this stage are captured, while others are incomplete, inconsistent, or lack experimental validation. NSCs, neural stem cells, ESCs, embryonic stem cells, iPSCs, induced pluripotent stem cells. Created with Biorender.com.

Despite their advantages as models of early neurogenesis, neural rosettes and EBs are limited in their ability to mimic neuronal migration, as detailed in Figure 2.

Neural rosettes provide a radial scaffold that allows partial directional migration (Grabiec et al. 2016) and EBs generally support only minimal and disorganized movement due to the lack of defined radial structures (Guy et al. 2021). In vivo studies using model organisms such as mice (Dudok et al. 2017) and zebrafish (Bingham et al. 2005) remain the gold standard for studying neuronal migration, not only due to their intact 3D tissue architecture that provides the physiological context needed for coordinated neuronal movement, but also because, in the case of mice, numerous mutant strains exhibit aberrant neuronal migration, which are particularly useful for studying neuronal migration since they reveal how specific genes and cellular mechanisms control this process in an intact 3D brain environment (Dudok et al. 2017). Neuronal movement is typically analyzed through live imaging which, besides having limited spatial resolution in deeper brain regions, is invasive (Azzarelli et al. 2017). Additionally, species‐specific differences also limit the extrapolation of findings to human neurogenesis (Azzarelli et al. 2017). In this context, brain organoids offer an experimentally accessible and ethically justifiable alternative for studying neuronal migration.

Brain organoids are 3D self‐organizing structures that, when exposed to appropriate biochemical cues and cultured under defined conditions (such as low‐adhesion environments, Matrigel embedding, and spinning bioreactors), undergo differentiation and spatial organization into structures that recapitulate key aspects of embryonic brain development (Azzarelli et al. 2017). Depending on the patterning signals and culture protocols used, organoids can mimic the whole brain or specific brain regions (including the cerebral cortex, hypothalamus, hippocampus, and cerebellum) where neurogenesis unfolds in a spatially and temporally regulated manner (Yang et al. 2022; Ajongbolo and Langhans 2025). Cortical brain organoids, in particular, have been extensively studied (Ajongbolo and Langhans 2025).

This system allows researchers to monitor the dynamic progression of neurogenesis, including the emergence of region‐specific transcriptional programs and cell–cell interactions that are difficult to capture in the previously mentioned culture configurations (Ajongbolo and Langhans 2025). Additionally, their relatively small size, reduced opacity, and simplified yet organized cytoarchitecture compared to in vivo tissue facilitate directional neuronal migration and its high‐resolution imaging (Aili et al. 2024). A side note, organotypic brain slices, which are an ex vivo model commonly derived from embryonic or early postnatal tissue, also provide a particularly useful platform for studying this process of neurogenesis (Koyama et al. 2012). Unlike brain organoids, these slices preserve the native cytoarchitecture, full cellular diversity, and extracellular matrix composition, maintaining the anatomical and connectivity differences between brain regions, though they reflect more mature brain stages (Nogueira et al. 2022). Although they can be used to study the migration of resident neurons, exogenous cells, such as pluripotent stem cell‐derived progenitors, can be introduced into these slices to assess migration trajectories and functional integration (Tanvig et al. 2009).

Despite their usefulness, organoids are also subject to several limitations that affect reproducibility and standardization. High batch‐to‐batch variability is a major concern, as different batches display significant morphological heterogeneity in size and shape, which affects tissue architecture and, consequently, cell differentiation (Aiello et al. 2025), with some cell populations reported to appear only in certain batches (Chen et al. 2019). This variability is further influenced by the differentiation strategy employed. Self‐patterned (unguided) organoids which are developed without external signaling cues tend to exhibit greater heterogeneity and spontaneously acquire regional identity, capturing a broader developmental diversity at the expense of higher variability. In contrast, growth factor–guided (patterned) protocols allow more reproducible results with a consistent generation of specific brain regions, but with less diversity (Chiaradia and Lancaster 2020). Another limitation arises from the lack of vascularization and diffusion constraints inherent to organoids, which result in hypoxia, nutrient deprivation, and accumulation of waste products, factors that cause necrotic centers and contribute to inconsistent differentiation outcomes (Urrestizala‐Arenaza et al. 2024), as detailed in Table 2. Furthermore, although brain organoids can replicate a rich diversity of cell types present in the native brain, they do not fully recapitulate its complete cellular milieu. Single‐cell transcriptomic studies have shown that organoids successfully generate multiple neuronal subtypes, as well as astrocytes and oligodendrocyte progenitors that can partially myelinate axons (Ajongbolo and Langhans 2025). However, several essential cell types remain underrepresented or absent, particularly those of non‐neuroectodermal origin, such as microglia and endothelial cells, which are crucial for immune responses, vascularization, and metabolic support (Chiaradia and Lancaster 2020; Ormel et al. 2018). But, although these models still cannot fully reproduce the complex cell interactions of the brain, they still provide a highly versatile and physiologically relevant platform for studying the cellular and molecular dynamics of neurogenesis (Chiaradia and Lancaster 2020).

Ultimately, the choice of the system used to culture pluripotent stem cells should be made based on the specific research question at hand, whether it requires controlled, reductionist conditions or a more complex and developmentally faithful model of neurogenesis.

## 3 Conclusions

The study of neurogenesis relies on a variety of cell models, each offering distinct advantages and limitations. These models are essential for unraveling the complex processes involved in neuronal development and function. However, selecting the most suitable approach should always be guided by research goals, practical considerations, and the available culture methods (Table 3), as these factors significantly influence research outcomes.

**TABLE 3:** Summary of the applicability of different cell models for specific neurogenesis‐related research applications. Applicable: Indicates established and well‐documented use; N/A: Denotes models biologically unsuitable for that aim.

Research aims	Primary brain cell cultures			Immortalized cell lines			Pluripotent stem cells (ESCs and iPSCs)		
	NSCs in neurospheres	Neurons in 2D	Neurons in 3D	SH‐SY5Y cells in 2D	SH‐SY5Y cells in 3D	IMR‐32 cells	Neural rosettes	Embryoid bodies	Brain organoids
Proliferation and self‐renewal	Applicable (Sathananthan 2011)	N/A	N/A	N/A	N/A	N/A	Applicable (Koch et al. 2009; Kim et al. 2024)	Applicable (Sato et al. 2016; Sathananthan 2011)	Applicable (Islam et al. 2024; Scholz et al. 2022)
Neuronal commitment and lineage progression	Applicable (Radecki and Samanta 2022)	N/A	N/A	Limited (differentiation protocols mimic some aspects of neuronal commitment) (Kovalevich and Langford 2013)	Limited (differentiation protocols mimic some aspects of neuronal commitment) (Kovalevich and Langford 2013)	Limited (differentiation protocols mimic some aspects of neuronal commitment) (Kovalevich and Langford 2013)	Applicable (Ziv et al. 2015; Zhou et al. 2016)	Applicable (Liyang et al. 2014; Luciani et al. 2024)	Applicable (Camp et al. 2015; van der Kroeg et al. 2024)
Migration	Limited (chain migration) (Koch et al. 2022; Imitola et al. 2004)	Limited (single cell migration, growth‐cone driven translocation) (Nakajima et al. 2024)	Limited (in scaffolds: single‐cell scaffold‐guided migration, in neurospheres: chain migration, in brain organoids: radial and tangential‐like migration) (Koch et al. 2022; Imitola et al. 2004; Pagan‐Diaz et al. 2020)	Limited (single cell migration) (Dwane et al. 2013)	Limited (single‐cell scaffold‐guided migration) (Dwane et al. 2013; Li, Livi, et al. 2007)	Limited (single cell migration) (Hiraiwa et al. 2019)	Limited (radial and interkinetic nuclear migration) (Ziv et al. 2015)	Limited (random migration) (Filipovic et al. 2012; Li et al. 2014)	Applicable (radial and limited tangential migration) (Park et al. 2025)
Morphology	Applicable (Koch et al. 2022; Espinoza et al. 2020)	Applicable (Banks et al. 2017)	Applicable (Hanson Shepherd et al. 2011)	Limited (depends on the differentiation protocol) (Fang et al. 2024)	Limited (depends on the differentiation protocol) (Agholme et al. 2010)	Limited (depends on the differentiation protocol) (Chaudhari et al. 2017)	Applicable (Herrera Lopez et al. 2025)	Applicable (Lilienberg et al. 2021)	Applicable (Hong et al. 2024)
Electrophysiology	Applicable (Pagani et al. 2006)	Applicable (best if cultured with other cells) (Goshi et al. 2023)	Applicable (Evans et al. 2019)	Applicable (depends on the differentiation protocol) (Santillo et al. 2014)	Applicable (depends on the differentiation protocol) (Innala et al. 2014)	Limited (depends on the differentiation protocol) (Rao and Kisaalita 2002)	Limited (developmental immaturity) (Innala et al. 2014)	Limited (depends on the differentiation protocol) (Pagan‐Diaz et al. 2020)	Applicable (Hong et al. 2024)
Synaptic and network potential	Applicable (Innala et al. 2014)	Applicable (Townshend et al. 2020)	Applicable (Innala et al. 2014)	Limited (useful to study only some aspects of synaptogenesis) (Kovalevich and Langford 2013)	Limited (useful to study only some aspects of synaptogenesis) (Kovalevich and Langford 2013; Franco‐Campos et al. 2025)	Limited (useful to study only some aspects of synaptogenesis) (Lekholm et al. 2021)	Limited (developmental immaturity) (Townshend et al. 2020)	Limited (depends on the differentiation protocol) (Townshend et al. 2020)	Applicable (Yakoub and Sadek 2019)
Scalability	Scalable potential (depends on protocol optimization) (Xiong et al. 2011)	Not scalable (Aggarwal et al. 2025)	Not scalable (Aggarwal et al. 2025)	Scalable (Kovalevich and Langford 2013)	Scalable (Kovalevich and Langford 2013)	Scalable (Leung et al. 2024)	Scalable (Frazier et al. 2025)	Scalable (Dang et al. 2004)	Scalable (Narazaki et al. 2025)
Ethical concerns	Yes (Pauly et al. 2018)			No (Frazier et al. 2025)			ESCs are not ethically sourced (Sugarman 2008), but iPSCs are (Hu et al. 2010)		

Primary cultures provide the advantage of maintaining many characteristics of neurons in vivo, making them more representative neuronal models compared to immortalized cell lines. Limitations regarding their use can be addressed by adopting different culture systems. For instance, cell population heterogeneity can be controlled using strategies like microfluidic chambers to separate neurons from other cells. However, some inherent limitations, such as their non‐human origin (which reduces their biological relevance to human systems) and the need to sacrifice animals to obtain these cultures, cannot be overcome by protocol optimization.

In this context, human neuroblastoma cell lines present a promising alternative due to their human origin. Their ability to acquire distinct neuronal phenotypes through manipulation of culture conditions highlights their remarkable flexibility. However, there is a critical need to optimize and standardize protocols to reliably differentiate these cells into specific neuronal‐like phenotypes in a controlled manner. Despite this flexibility, the fact that these cell lines are not actual neurons limits their biological relevance. Additionally, although they are typically used to model the differentiation of neurons, it remains challenging to determine which stage of neurogenesis these cell lines accurately replicate.

Human pluripotent stem cells, such as ESCs and iPSCs, address many limitations of both primary cultures and neuroblastoma cell lines. They have the advantage of a human origin combined with the ability to generate fully differentiated, functional neurons. These cells, depending on their culture method, have the potential to replicate almost all stages of neurogenesis, from an undifferentiated state to mature neuronal cells, allowing researchers to study each step of neurogenesis in detail. However, they also pose challenges: their complexity and the lack of standardized protocols currently limit their widespread use compared to simpler systems like primary cultures or neuroblastoma cell lines.

Overall, this work aimed to contribute to a better understanding of the available options for studying neurogenesis in vitro and to provide a comprehensive overview of their respective strengths and drawbacks. Throughout this manuscript, it becomes clear that no single model is ideal, and while each one of the reviewed models provides valuable critical mechanistic insights, complementary in vivo studies remain essential to confirm the physiological relevance of in vitro findings. Ultimately, making informed decisions based on specific research goals, the nature of the scientific question, and the resources available is crucial to advancing our understanding of neurogenesis and its underlying mechanisms.

## Author Contributions

**Mariana Vassal:** writing – original draft, visualization, investigation, conceptualization. **Ana C. Cruz:** writing – original draft, visualization. **Sandra Rebelo:** conceptualization, funding acquisition, writing – review and editing, supervision. **Filipa Martins:** conceptualization, funding acquisition, writing – review and editing, supervision.

## Funding

Open access funding provided by FCT|FCCN (b‐on). This work was supported by national funds, through Fundação para a Ciência e Tecnologia of the Ministério da Educação e Ciência (FCT/MCTES) [grant number PTDC/BTM‐TEC/3792/2021], and by the Institute of Biomedicine (iBiMED) under Grant UID 4501‐ Instituto de Biomedicina—Aveiro. Mariana Vassal and Ana Cruz were recipients of FCT Studentships, grant number 2023.01360.BD, and 2024.03977.BD, respectively.

## Ethics Statement

The authors have nothing to report.

## Consent

The authors have nothing to report.

## Conflicts of Interest

The authors declare no conflicts of interest.

## Data Availability

Data sharing not applicable to this article as no datasets were generated or analysed during the current study.
